# Oxidative stress biomarkers are associated with visible clinical signs of a disease in frigatebird nestlings

**DOI:** 10.1038/s41598-017-01417-9

**Published:** 2017-05-09

**Authors:** Manrico Sebastiano, Marcel Eens, Hamada Abd Elgawad, Benoît de Thoisy, Vincent Lacoste, Kévin Pineau, Han Asard, Olivier Chastel, David Costantini

**Affiliations:** 1 Behavioural Ecology and Ecophysiology group, Department of Biology, University of Antwerp, Universiteitsplein 1, 2610 Wilrijk, Belgium; 2 Integrated Molecular Plant Physiology Research, Department of Biology, University of Antwerp, Groenenborgerlaan 171, 2020 Antwerp, Belgium; 3 Faculty of Science, Department of Botany and Microbiology, University of Beni-Suef, Beni-Suef, Egypt; 4 Laboratory of Virus-Host Interactions, Institut Pasteur de la Guyane, Cayenne, French Guiana France; 5 Groupe d’Etude et de Protection des Oiseaux en Guyane (GEPOG), 15 Avenue Pasteur, 97300 Cayenne, French Guiana France; 6 Centre d’Etudes Biologiques de Chizé (CEBC), UMR7372- CNRS/University of La Rochelle, F-79360 Villiers-en-Bois, France; 7 Institute of Biodiversity, Animal Health and Comparative Medicine, School of Life Sciences, University of Glasgow, Graham Kerr Building, Glasgow, G12 8QQ UK; 8 UMR 7221, Muséum National d’Histoire Naturelle, 7 rue Cuvier, 75231 Paris Cedex 05, France

## Abstract

Infectious diseases are one of the most common threats for both domestic and wild animals, but little is known about the effects on the physiological condition and survival of wild animals. Here, we have tested for the first time in a wild vertebrate facing a viral disease possibly due to herpesvirus (i) whether nestlings with either low levels of oxidative damage or high levels of antioxidant protection are less susceptible to develop visible clinical signs, (ii) whether the disease is associated with the nestlings’ oxidative status, (iii) whether the association between the disease and oxidative status is similar between males and females (iv), and whether cloacal and tracheal swabs might be used to detect herpesvirus. To address our questions, we took advantage of a population of Magnificent frigatebirds (*Fregata magnificens*) whose nestlings have experienced high mortality rates in recent times. Our work shows that (i) blood lipid oxidative damage is associated with observable clinical signs and survival probabilities of nestling frigatebirds, and (ii) that high glutathione levels in red blood cells are associated with the emergence of visible clinical signs of the disease. Our work provides evidence that differences in the oxidative status of nestlings might underlie individual health and survival.

## Introduction

Wildlife infectious diseases have enormous ecological and public health impacts^1^. Notorious examples of infectious diseases that are dramatically causing massive population declines are the fungus *Batrachochytrium dendrobatidis* that is affecting frogs worldwide^2^ or a novel infectious cancer that is jeopardizing the future of the Tasmanian devil *Sarcophilus harrisii* ^3^. Viruses also represent a serious threat for viability of wild animal populations^2, 4^. Among them, herpesviruses are widespread throughout the biosphere and are one of the most common viral agents in wild and domestic animals^5^, with various clinical manifestations in several avian species throughout the world^6^, and been found in many animal species^7^. Herpesviruses are generally only recognized when they cause visible clinical signs, such as skin crusts^8^. In addition, papilloma viruses are also only recognized when they cause the appearance of clinical signs, as the cutaneous lesions, as those recently described in the Cape mountain zebra *Equus zebra zebra*, the giraffe *Giraffa camelopardalis*, the sable antelope *Hippotragus niger*, and the African buffalo *Syncerus caffer* in South Africa^9, 10^. It is therefore likely that an individual might be carrying the pathogen without any visible manifestation of the disease, until exposure to an environmental stressor can trigger activation of viral replication^6^. Although many studies have assessed the impact of viruses and parasites on physiology and mortality of domestic animals^11–15^, data are scarce for wild animals facing viral diseases^16–20^.

Exposure to sources of environmental stress (e.g., food shortage) can decrease individual immunocompetence, facilitating viral activation and occurrence of clinical signs of viral diseases^21, 22^. For instance, it has been previously shown that the exposure to contaminants increases the susceptibility of mice to *Leishmania* parasites^23^. At a molecular level, some clinical studies have suggested that oxidative stress (OS), which is defined as “the rate at which oxidative damage is generated”^24^, might be the physiological mechanism that promotes virus activation^25–27^, thus linking environmental stress to the occurrence of the disease. Furthermore, over the last decade there has been an increasing interest in OS as one mechanism mediating life history trade-offs^28, 29^. The cell oxidative status represents a complex balance between pro-oxidant and antioxidant molecules^29^. When organisms are exposed to a source of stress, increased generation of reactive oxygen species (ROS) may lead to an unbalanced oxidative status in favour of oxidant molecules, resulting in increased production of molecular oxidative damage^30^. As with viral diseases, OS may make cells more susceptible to viral activation and replication^31^. This potential association between OS and viral activation is further supported by several studies, which have found that administration of antioxidants may reduce the oxidative damage and the viral burden^32, 33^. Specifically, a recent meta-analysis across many different domestic and animal species has provided support for the hypothesis that OS is likely to be one molecular mechanism responsible of pathological effects of herpesvirus infection^34^. This meta-analysis showed that herpesvirus infection decreases the level of non-enzymatic antioxidants, increases the generation of reactive oxygen species (ROS) and causes oxidative damage to biomolecules^34^. It is thus possible that herpesviruses modify the oxidative status of the host promoting its replication. For instance, oxidative damage to cells may facilitate herpesvirus permissiveness in the nervous system^35^, and OS is known to activate the nuclear factor-kappa B (NF-*k*B), which participates in the regulation of the herpesvirus replication cycle^36^. Furthermore, since NF-*k*B seems to be a key factor associated with viral infections^37^, the role of oxidative stress as a regulator of the viral activation and as a pathogenic factor associated with infections has been recently investigated^38, 39^.

**Figure 1:**
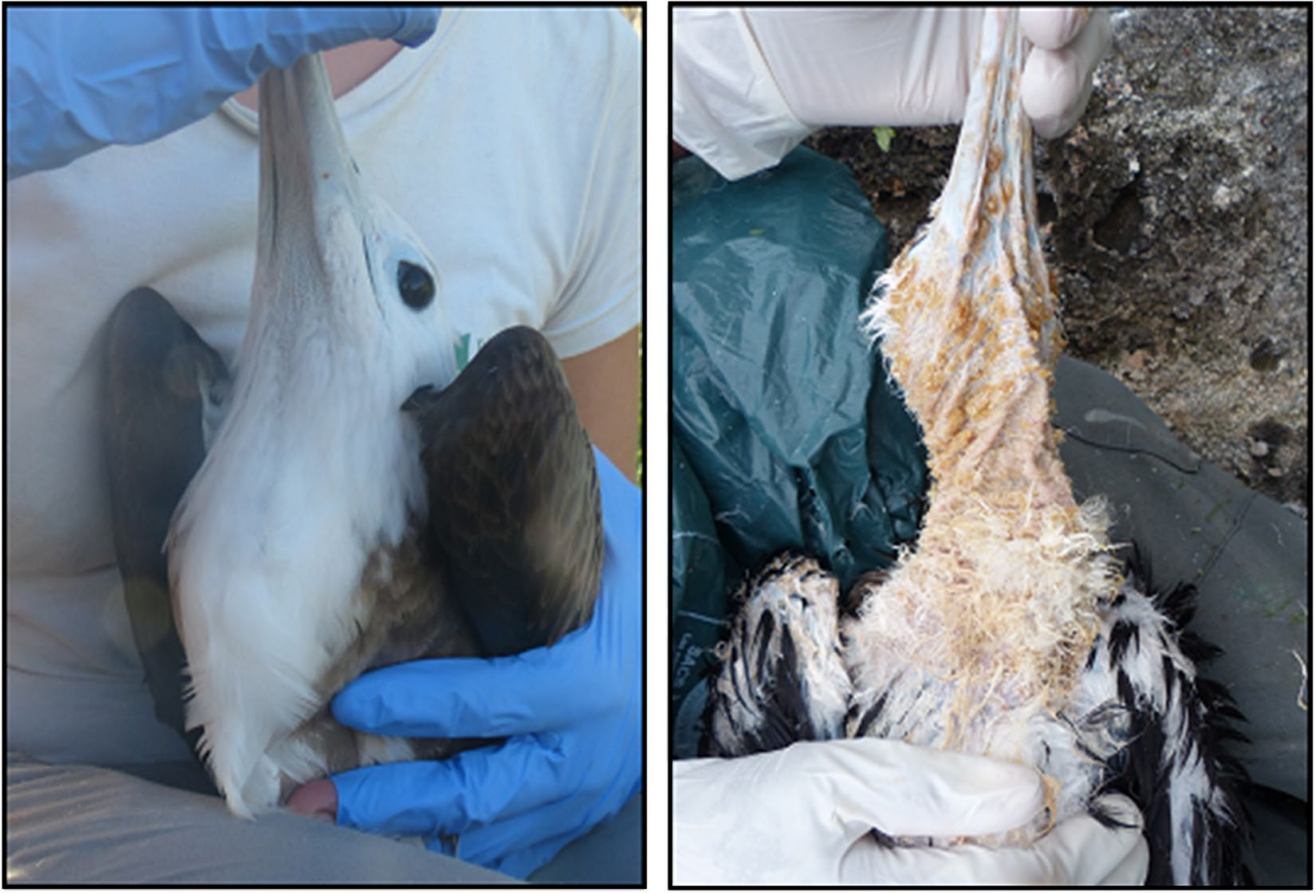
A healthy nestling (on the left) does not show clinical signs (hyperkeratosis, body and head crusts) in comparison to a sick individual (on the right).

Although some studies have found OS to be associated with infectious diseases and possibly to be one mechanism responsible for the patho-physiological consequences of several viral infections^26, 34, 40^, (i) most of the work has been done on humans and laboratory or domestic animals; and (ii) the role of oxidative stress in determining health and survival perspectives is complex and results have often been contradictory^28, 29^. These seemingly contradictory results can also be found in studies on viral diseases, possibly because of the trade-off between the costs and benefits associated with the increase in oxidative stress during infections^41^. For example, the organism may first respond to an infection by increasing the production of ROS from immune cells (the so-called oxidative burst) in order to eliminate the infectious agent^41^, but these non-specific compounds are also harmful to the host’s tissues^42^. Hence, it is essential to assess whether the individual oxidative status is associated with the occurrence of clinical signs and how this status is modified during the progress of the infection. To this end, we carried out a study on oxidative stress, clinical signs, and survival probabilities in nestlings of a tropical seabird, the Magnificent frigatebird (*Fregata magnificens*, Mathews 1914, hereafter frigatebirds) facing a severe infectious disease. A previous study carried out on this population breeding on Grand Connétable island, in French Guiana, excluded the possibility of a bacterial infection and the presence of ectoparasites after bacterial cultures and microscopic evaluation of skin samples^43^. Bird samples were further tested and gave negative results for avian poxvirus DNA but were positive to herpesvirus DNA^43^, which therefore seems the possible causal agent responsible for the appearance of clinical signs as illustrated in Fig. 1, which are only noticeable in nestlings. Visible clinical signs of the disease occur in most nestling during their development, and their chances of survival are extremely low (5 to 15% accordingly to field observations).

Given that the observed clinical signs are likely related to an infectious disease, this frigatebird population is a relevant biological model for investigating the relationships among oxidative stress, clinical signs of the disease and survival probabilities. In addition, since previous studies showed a sex-related response to oxidative stress^44, 45^, we have also evaluated sex differences in OS biomarkers. Specifically, the aims of this study were to investigate (i) whether nestlings with either low levels of oxidative damage or high levels of antioxidant protection are less susceptible to develop visible clinical signs, (ii) whether the disease is associated with the nestlings oxidative status, (iii) whether the impact of the disease on oxidative status and survival is similar between males and females (iv), and whether the use of cloacal and tracheal swabs might be used as a non-invasive tool to detect the presence of the infectious agent in individuals without clinical signs. Our study is also relevant for conservation because it may provide conservation practitioners with tools to predict the impact of a pathogen on fitness traits.

## Material and Methods

### Ethics statement

All activities were performed in accordance with the relevant guidelines and regulation and all experimental protocols were approved by the Préfet de la Région Guyane (Direction de l’Environnement, de l’Aménagement et du Logement – Service Milieux Naturels, Biodiversité, Sites et Paysages – Permit number 2015131-0022).

### Sample collection

The fieldwork was carried out in 2015 on Grand Connétable island, a protected area located off the Northern Atlantic coast of South America (French Guiana, 4°49′30 N; 51°56′00 W). This small island hosts a unique colony of Magnificent frigatebirds that is one of the most important in South America, and represents the only breeding site for frigatebirds in French Guiana^46^. A total of 44 four-months old nestlings including 22 nestlings without clinical signs and 22 sick nestlings showing severe clinical signs were randomly chosen at different sites of the island. The selection of individuals was simplified by the absence or presence of visible clinical signs (Fig. 1), and healthy nestlings were carefully checked for detectable clinical signs of the disease (e.g., little crusts hidden under their plumage). All individuals were captured by hand on June 5^th^–7^th^. On June 19^th^–21^th^, the same birds were captured and sampled again, in order to be able to evaluate whether the physiological status of nestlings changed during the progression of the disease. A period of about two weeks of delay from the first to the second sampling period has been chosen given that field observations suggested that this is the timeframe required to cause observable clinical signs of the disease. In the second sampling period, of the nestlings that were previously blood-sampled nine were found dead from the disease (one healthy and eight sick nestlings), and four were not found (of which all of them were healthy). Thus, six new nestlings were sampled to increase the number of individuals at the second sampling period in order to perform statistical comparisons among groups. Out of the 22 healthy individuals sampled during the first period, six nestlings showed the occurrence of clinical signs in the second period (body and head crusts, hyperkeratosis on eyes with consequent thickening of the cornea). Despite frigatebird nests occur at a high density on this island, a previous experiment on pigeons has shown how horizontal transmission of herpesvirus (from one bird to another) is a rare event, while it seems more likely that herpesvirus is transmitted vertically (from parents to offspring)^6^.

Within 3 minutes after capture, two mL of blood were collected from the brachial vein using a heparinized syringe and a 25 G needle. Samples were immediately kept cold and centrifuged in the field within less than one hour to separate plasma and red blood cells (used for oxidative stress biomarkers). Both samples of plasma and red blood cells were then kept in dry ice until the end of the fieldwork and, when back to the laboratory, were kept in a −80 °C freezer until laboratory analyses. An aliquot of blood was also used for sex determination of nestlings according to a previous protocol with minor modifications^47^.

In addition to blood samples, cloacal and tracheal cotton swabs were collected from 20 nestlings during the first sampling period (10 nestlings with clinical signs and 10 looking-healthy nestlings), and 21 nestlings during the second sampling period (11 nestlings with clinical signs and 10 looking-healthy nestlings), respectively. Since previous laboratory analysis suggested that clinical signs were likely to be related to a herpesvirus infection^43^, swab samples were taken to test whether herpesvirus is also detectable in tracheal and cloacal cavities of healthy individuals. Indeed, a previous study showed that healthy individuals of different bird species might excrete virus particles in the faeces and might show persistency of herpesvirus in the pharynx, even when the virus is latent^6^. Both swab and blood samples were stored at −80 °C until laboratory analyses.

### Analyses of metrics of oxidative status

All analyses were done using established protocols for vertebrates. High performance liquid chromatography (HPLC) with electrochemical detection was applied for determination of the non-enzymatic antioxidant capacity using reduced (GSH) and oxidized (GSSG) glutathione in red blood cells by Reversed-Phase HPLC of Shimadzu (Hai Zhonglu, Shanghai) according to a previous protocol^48^. Concentrations were expressed as μmol/g of fresh weight. The GSH/GSSG ratio was also calculated and used as a metric of oxidative balance^49^. The enzymatic antioxidant capacity was measured using three different biomarkers. Superoxide dismutase (SOD) activity was determined in red blood cells by measuring the inhibition of nitroblue tetrazolium reduction at 560 nm and was expressed as U/mg protein per minute^50^. Catalase activity (CAT) was assayed in red blood cells by monitoring the rate of decomposition of H_2_O_2_ at 240 nm and was expressed as μmol H_2_O_2_/mg protein per minute^51^. Glutathione peroxidase (GPX) activity was measured in red blood cells by a spectrophotometric method and was expressed as μmol NADPH/mg protein per minute^52^. The non-enzymatic antioxidant capacity of plasma was quantified using the OXY absorbent test (Diacron International, Grosseto, Italy) and was expressed as mM HOCl neutralized^53^. Plasma lipid peroxidation was quantified using the Thiobarbituric Acid Reactive Substances (TBARS) assay; values were expressed as nmol of Malondialdeyde (MDA) equivalents/mL of plasma^54^. Oxidative protein damage was determined by measuring protein carbonyls in red blood cells; values were expressed as nmol/mg protein^55^. Detailed protocols can be found in the supplementary material.

### Assessment of the presence of the virus in swabs and crusts

Swabs and crusts were suspended in separate tubes in 600 µl of Dulbecco's Modified Eagle Medium (DMEM) for 30 minutes, then vortexed and centrifuged for a few seconds. Total DNA was extracted using the NucliSENS easyMAG bio-robot (bioMérieux, Marcy l’Etoile, France). Molecular screening targeted a fragment of the *DNA polymerase gene* previously described in Frigatebirds^43^, with a semi-nesting PCR approach. The first PCR was done with primers F1S (5′-TGCTGAGCGTTTTGTTGC-3′) and FAS (5′-TGTTCCTTCCTATGGTCGTTAC-3′) and the following amplification conditions: 10 min at 94 °C, followed by 35 cycles of 30 sec at 94 °C, 30 sec at 57 °C, 30 sec at 72 °C, and a final extension of 10 min at 72 °C. The PCR product (expected size 212 bp) was then used as template for a semi-nested PCR with primers F2S (5′-GCGGTTTTGCTGGACAAG-3′) and FAS, and the following amplification profile: 10 min at 94 °C, followed by 35 cycles of 30 sec at 94 °C, 30 sec at 60 °C, 30 sec at 72 °C, and a final extension of 10 min at 72 °C. The PCR product was 132 bp, samples were deposed and revealed on an agarose gel.

Quantitative real-time polymerase chain reaction detection of Frigatebird herpesvirus was performed on tracheal and cloacal swabs collected at the first sampling period (n = 20) by using a TaqMan technique from 100 ng DNA extracted from cloacal and tracheal cotton swabs. The primers and probe set were designed in the *DNA polymerase* gene fragment (FregF1: 5′-GCACGTTAGGGAGAGCTTGCT-3′; FregR1: 5′-GCGCATCGCCAACCA-3′; Freg-MGB probe: 5′-AGCGTTTTGTTGCGCG-3′) (Eurogentec, Belgium). Quantitative real-time PCR assay was performed on a StepOne Plus apparatus (Applied Biosystems) using components supplied in the TaqMan universal master mix II (Thermo Fischer Scientific) in a reaction volume of 25 μL. Thermal cycling was initiated with 2 minutes of incubation at 50 °C, followed by 10 minutes of denaturation at 95 °C, and then 40 cycles of 95 °C for 15 seconds and 60 °C for 1 minute. A standard curve was based on PCRs performed twice in duplicate by using DNA copy numbers ranging from 2 to 2 × 10^7^ copies calculated from a relevant cloned plasmid containing the same Frigatebird herpesvirus *DNA polymerase* gene sequence. The cycle threshold values were plotted against given copy numbers and show a linear amplification between 2 × 10^2^ and 2 × 10^7^ copies.

### Sex determination

DNA samples were used to determine the sex of individuals by polymerase chain reaction amplification as previously detailed with minor modifications^47^. Sex identification was carried out using the P8 (5′-CTCCCAAGGATGAGRAAYTG-3′) and P2 (5′-TCTGCATCGCTAAATCCTTT-3′) primers. An initial denaturing step at 94 °C for 2 min was followed by 40 cycles of 94 °C for 30 s, 48 °C for 30 s, and 72 °C for 1 min. A final run of 94 °C for 30 s, 50 °C for 1 min, and 5 min at 72 °C completed the program.

### Statistical analysis

All statistical analyses were performed using R (v. 3.1.2).

Linear mixed models were used to assess whether the biomarkers of oxidative status differed among groups and changed with sampling period. In each model, a given biomarker was included as dependent variable; group and sampling period were included as fixed factors and the factor “individual” was included as a random effect because we had repeated measurements. Outcomes of all models were unchanged if the sampling time (calculated as minutes elapsed since midnight) was included. Thus, we presented outcomes of models that do not take into account sampling time. In each model, we also included the interaction between the group and the sampling period. All individuals for which we had repeated measurements (data from both the first and the second period) were included (for a total of 31 individuals). These 31 individuals were in three different groups prior to statistical analysis. A first group included nestlings that did not show any visible sign of the disease in both sampling periods (hereafter called “HH”, n = 11). A second group included nestlings that manifested clinical signs only in the second sampling period (hereafter called “HS”, n = 6). A third group included nestlings that already showed clinical signs during the first sampling period (hereafter as “SS”, n = 14). This approach has been chosen in order to account for differences among groups during the first sampling, as well as to determine the “change” (a significant decrease or increase in the specific biomarker) of each group from the first to the second period. Furthermore, an additional model including the same factors as those of the previous one was carried out including only the individuals sampled at the first period. This has been done to avoid the possibility of a bias in the model due to the selective disappearance of the dead nestlings (that were therefore not included in the model with repeated measurements), as 8 out of the 9 birds that died were from the sick group. In this model, in addition to the HH group (which now includes the individual who died and four that were not found at the second period, n = 16), HS group (n = 6), SS group (n = 14), an additional group was therefore included (hereafter “SD”, n = 8). From each model, we first removed the interaction term when it was not significant and then the non-significant fixed factors, in order to attain the best-fit model. Finally, we applied a post-hoc comparison based on the difference of least square means.

In order to evaluate sex differences, the whole dataset has been divided in two different datasets: one for the first sampling period (n = 44; 22 nestlings without clinical signs and 22 with clinical signs) and one for the second sampling period in order to include individuals that have been sampled only in one period (n = 37; 31 re-captured plus six new individuals). Then, we have also included the interaction between sex and group in the model to be able to detect sex differences within the sick and the healthy groups. Dependent variables were tested for normality with a Shapiro-Wilk and were log-transformed when necessary (with the exception of TBARS that showed a deep difference among groups and for which a normalization was not possible). Models were also tested for heteroscedasticity and residual normality. For each model we removed the non-significant interaction first, and then each non-significant factor in order to get to the best fit model. Finally, we performed multiple comparisons with the glht function in R (post-hoc analysis: “multcomp” package).

A generalized linear model using a logit link function and a binomial error variance was used to assess if any metric of oxidative status was associated with either the occurrence of clinical signs or the mortality of nestlings. The prediction of viral activation was estimated from each oxidative stress biomarker in the 22 healthy individuals in the first period. The survival perspectives within our study period were estimated using two different models: one on the 22 sick individuals sampled during the first period and the other one on the 44 individuals sampled during the first period. In these models, an estimation of overdispersion has been performed to minimize the risk of Type I error^56^.

Finally, Principal Component Analysis on a correlation matrix among the different markers of oxidative status was done to reduce the number of variables. Generalized linear models were then used to test whether these new variables were associated with either the occurrence of clinical signs or mortality of nestlings. This approach enabled us to assess whether, as compared to single markers, variables integrating information from multiple markers are better to describe the progress of the disease.

Any violations of model assumptions, and transformation of data are reported when needed.

## Results

### Analysis of viral DNA

Herpesviral DNA was detected in the tracheal and cloacal swabs of seven out of ten healthy-looking individuals sampled during the first period, compared to nine out of ten individuals showing clinical signs at the first sampling period. In the second sampling period, all the healthy-looking nestlings (ten) were positive, and six out of eleven nestlings with clinical signs were positive to at least one swab. The three healthy individuals that did not reveal presence of the virus in swabs during the first sampling period showed clinical signs of infection in the second sampling period, *i*.*e*. two weeks later.

The use of the qPCR identified the presence of viral DNA from low copies up to 10^6^ to 10^7^ copies in tracheal swabs of six out of ten sick individuals at the first sampling period, which were either found dead (three) or in a critical health state (three) in the second sampling period. Viral DNA was also detected in tracheal swabs of five out of ten healthy individuals sampled during the first sampling period. In those five individuals, viral copies were found at lower levels (from a low number of copies up to 10^4^ to 10^5^ copies), than in sick nestlings, equivalent to a difference of millions of viral copies. In the second sampling period, among them, two were not found and two nestlings showed the occurrence of clinical signs of the disease. Finally, the virus was undetectable by qPCR on DNA extracted from cloacal swabs in most tested individuals (60%). For the only few individuals for which the virus was present (40%), there were far less copy numbers (<300 copies) in comparison to tracheal swabs. Detailed results of the two PCR approaches can be found in Supplementary Table S1.

### Non-enzymatic antioxidants

**Figure 2:**
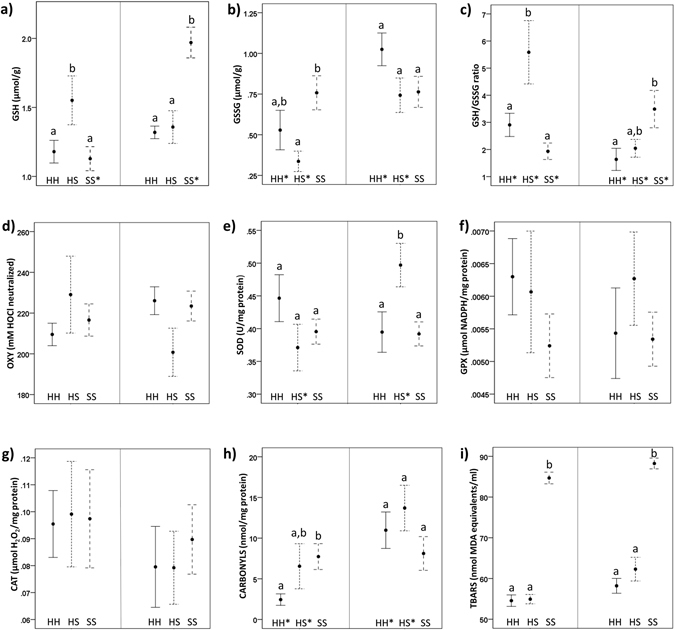
Mean and standard error of the biomarkers of OS. Different groups are indicated on the x axis by the acronyms “HH” (n = 11), “HS” (n = 6), and “SS” (n = 14). After post-hoc comparisons on the GSH, GSSG, GSH/GSSG, SOD, CARBONYLS, and TBARS models, plots which share the same letter showed no significant differences within the first period (left panel), or the second sampling period (right panel). Groups with an asterisk next to their acronym had a significant increase or decrease from the first to the second period.

The model for GSH showed a significant interaction between group and sampling period (*F* = 19.02, *P* < 0.01). In the first period, the HS group had higher GSH levels than both the HH group (*t* = −2.25, *P* = 0.03) and the SS group (*t* = 2.66, *P* = 0.01), while in the second period the SS group had higher GSH levels than both the HH group (*t* = −4.95, *P* < 0.01) and the HS group (*t* = −3.84, *P* < 0.001). The SS group had a strong increase in GSH levels from the first to the second period (*t* = −8.22, *P* < 0.01) (Fig. 2a, Table 1).

**Table 1:** Full and best fit linear mixed models of nine biomarkers of non-enzymatic antioxidants, enzymatic antioxidant activity, and oxidative damage, respectively.

		Initial model			Final model		
		*df*	*F*-value	*P*-value	*df*	*F*-value	*P*-value
GSH	Group	2,28	4.02	**0.03**			
	Period	1,28	12.83	**<0.01**			
	Group * Period	2,28	19.02	**<0.01**			
GSSG	Group	2,28	1.96	0.16			
	Period	1,28	10.87	**<0.01**			
	Group * Period	2,28	3.57	**0.04**			
GSH/GSSG	Group	2,28	2.36	0.11			
	Period	1,28	5.53	**0.03**			
	Group * Period	2,28	12.06	**<0.01**			
OXY	Group	2,28	0.13	0.88	2,28	0.13	0.88
	Period	1,28	0.05	0.82	1,30	0.23	0.64
	Group * Period	2,28	2.73	0.08			
SOD	Group	2,28	0.79	0.46			
	Period	1,28	1.44	0.24			
	Group * Period	2,28	5.91	**<0.01**			
GPX	Group	2,28	0.92	0.41	2,28	0.92	0.41
	Period	1,28	0.16	0.70	1,30	0.25	0.62
	Group * Period	2,28	0.57	0.57			
CAT	Group	2,28	0.09	0.91	2,28	0.09	0.91
	Period	1,28	1.07	0.31	1,30	1.01	0.32
	Group * Period	2,28	0.07	0.93			
CARBONYLS	Group	2,28	1.10	0.35			
	Period	1,28	10.76	**<0.01**			
	Group * Period	2,28	5.63	**<0.01**			
TBARS	Group	2,28	200.49	**<0.01**	2,28	200.49	**<0.01**
	Period	1,28	14.10	**<0.01**	1,30	12.88	**<0.01**
	Group * Period	2,28	0.75	0.48			

Significant P-values are bolded. When a final model is not shown, initial and final models matched.

The model for GSSG showed a significant interaction between group and sampling period (*F* = 3.57, *P* = 0.04). In the first period, the SS group had more GSSG than the HS group (*t* = −2.47, *P* = 0.02), while there were no differences between groups in the second period. GSSG significantly increased from the first to the second period in the HS (*t* = −2.07, *P* = 0.05) and in the HH group (*t* = −3.42, *P* < 0.01) (Fig. 2b, Table 1).

The model for log-transformed GSH/GSSG ratio showed a significant interaction between group and sampling period (*F* = 12.06, *P* < 0.01). In the first period, the HS group had higher values of GSH/GSSG ratio than both the HH group (*t* = −2.23, *P* = 0.03) and the SS group (*t* = 3.90, *P* < 0.01), while in the second period the GSH/GSSG ratio was higher in the SS group than in the HH group (*t* = −3.06, *P* < 0.01). The GSH/GSSG ratio decreased from the first period to the second period in the HH group (*t* = 2.75, *P* = 0.01) and in the HS group (*t* = 3.13, *P* < 0.01), while it increased in the SS group (*t* = −2.83, *P* < 0.01) (Fig. 2c, Table 1).

The non-enzymatic antioxidant capacity of the plasma (OXY test) did not differ among groups, nor did it show any significant changes over time (*F* < 2.73, *P* > 0.08) (Fig. 2d, Table 1).

### Enzymatic antioxidant activity

SOD showed a significant interaction between group and sampling period (*F* = 5.91, *P* < 0.01). In the first period there were non-significant differences between groups. SOD activity showed a significant increase in the HS group over time (*t* = −3.01, *P* < 0.01), so that the SOD activity in the HS group became higher than both the HH group (*t* = −2.26, *P* = 0.03) and the SS group (*t* = 2.42, *P* = 0.02) (Fig. 2e, Table 1). GPX and CAT activities did not differ among groups, nor did they change significantly over time (*F* < 0.92, *P* > 0.41 and *F* < 1.07, *P* > 0.31, respectively) (Fig. 2f and 2g, Table 1).

### Oxidative damage

The model for log-transformed protein carbonyls showed a significant interaction between group and sampling period (*F* = 5.63, *P* < 0.01). In the first period, the HH group had less carbonyls than the SS group (*t* = −3.02, *P* < 0.01), while there were no differences between groups in the second period. Carbonyls significantly increased in the HH group (*t* = −3.87, *P* < 0.01) and in the HS group (*t* = −2.08, *P* = 0.04) from the first to the second period (Fig. 2h, Table 1).

The model for TBARS showed significant differences among groups (*F* = 200.49, *P* < 0.01) and between periods (*F* = 14.10, *P* < 0.01) but the interaction between group and sampling period was not significant (*F* = 0.75, *P* = 0.48). The SS group had higher levels of TBARS than both the HH group (*t* = −18.39, *P* < 0.01) and the HS group (*t* = −14.06, *P* < 0.01), while there were no significant differences between the HH group and the HS group. Finally, TBARS generally increased over time (*t* = −3.59, *P* < 0.01) (Fig. 2i, Table 1).

### Sex differences in oxidative status

In the first sampling period, males and females had similar values for all the analyzed biomarkers (LM: *F* < 2.3, *P* > 0.13), with the exception of the OXY values (*F* = 5.95, *P* = 0.02). Females had higher OXY values than males (*t* = −2.50, *P* < 0.02). In the second sampling period, males and females did not differ in any of the measured biomarkers (*F* < 2, *P* > 0.17).

### Differences at the first sampling period

No differences were found among the HH, HS, SS and SD groups in the models on GSH, OXY, SOD, GPX and CAT (Figure S1).

The model for log-transformed GSSG values showed significant differences among groups (*F* = 4.81, *P* < 0.01, Figure S1b), with the SS group showing higher values than the HS group (*t* = 3.19, *P* = 0.01).

The model on log-transformed GSH/GSSG ratio showed significant differences among groups (*F* = 6.31, *P* < 0.01, Figure S1c). The HS group showed higher GSH/GSSG ratio than both the SS group (*t* = −3.93, *P* < 0.01) and the SD group (*t* = −3.17, *P* = 0.02), while the HH group had higher values than the SS group (*t* = −2.70, *P* < 0.05).

Protein carbonyls also differed among groups (*F* = 2.87, *P* < 0.05, Figure S1h), with the SS group having higher content of protein carbonyls than the HH group (*t* = 2.87, *P* = 0.03).

Finally, TBARS levels were significantly different among groups (*F* = 122.71, *P* < 0.01, Figure S1i). The SD group had higher concentration than both the HH (*t* = 12.67, *P* < 0.01) and the HS group (*t* = 10.10, *P* < 0.01), while the SS group had higher concentration than both the HH (*t* = 16.27, *P* < 0.01) and the HS group (*t* = 12.13, *P* < 0.01).

### Nestling oxidative status as predictor of viral activation and short-term survival

**Figure 3:**
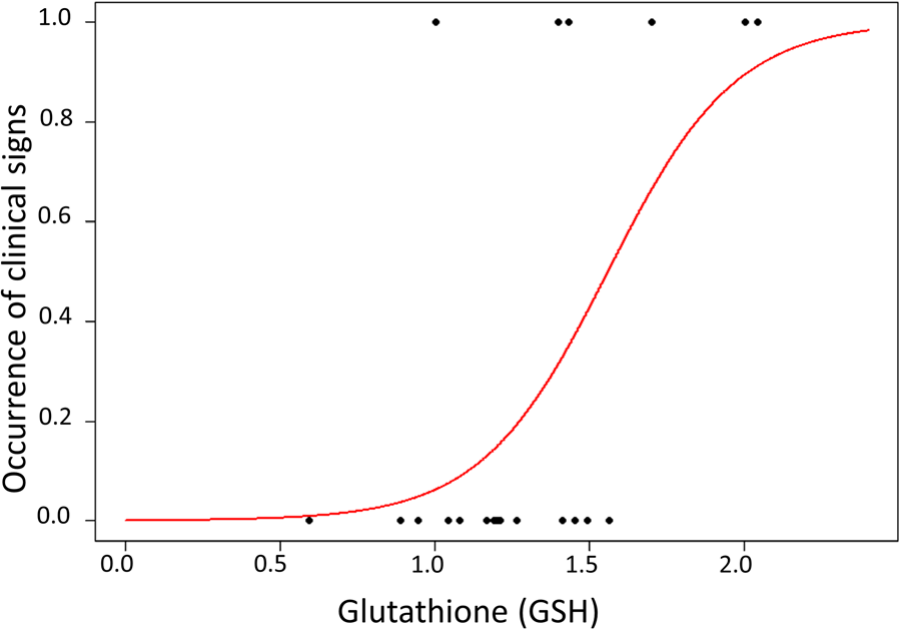
Nestling probability to show clinical signs (sick = 1 and healthy = 0) over a sixteen days period in relation to GSH expressed as μmol/g of fresh weight.

**Figure 4:**
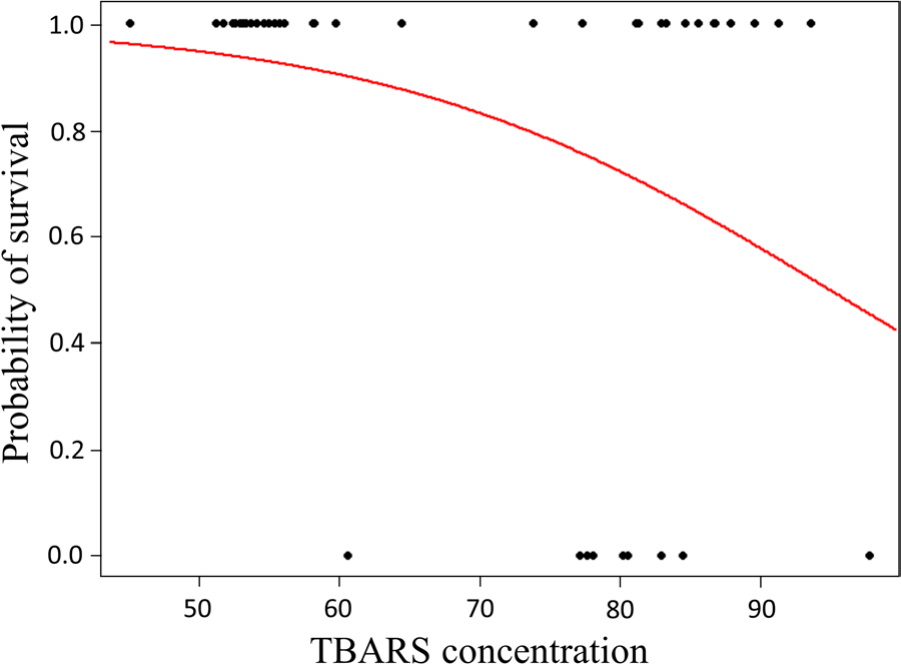
Survival probability over a sixteen days period of nestlings (alive = 1 and dead = 0) in relation to the TBARS expressed as nmol MDA equivalent/ml.

We found a positive and significant covariation between GSH concentration and the probability of the occurrence of clinical signs (Fig. 3). HS nestlings had significantly higher levels of GSH as compared to HH nestlings (GLM: *Z* = 2.0, *P* < 0.05), while none of the other biomarkers were associated with the occurrence of clinical signs (GLM: *Z* < 1.8, *P* > 0.09). In addition, none of the considered biomarkers in this study were associated with short-term survival in SS nestlings (GLM: *Z* < 1.7, *P* > 0.09). Conversely, TBARS was associated with short-term survival (with higher values indicating lower survival perspectives) when all nestlings were included in the model (GLM: *Z* = −2.1, *P* = 0.03; Fig. 4).

### PCA of oxidative status biomarkers

**Figure 5:**
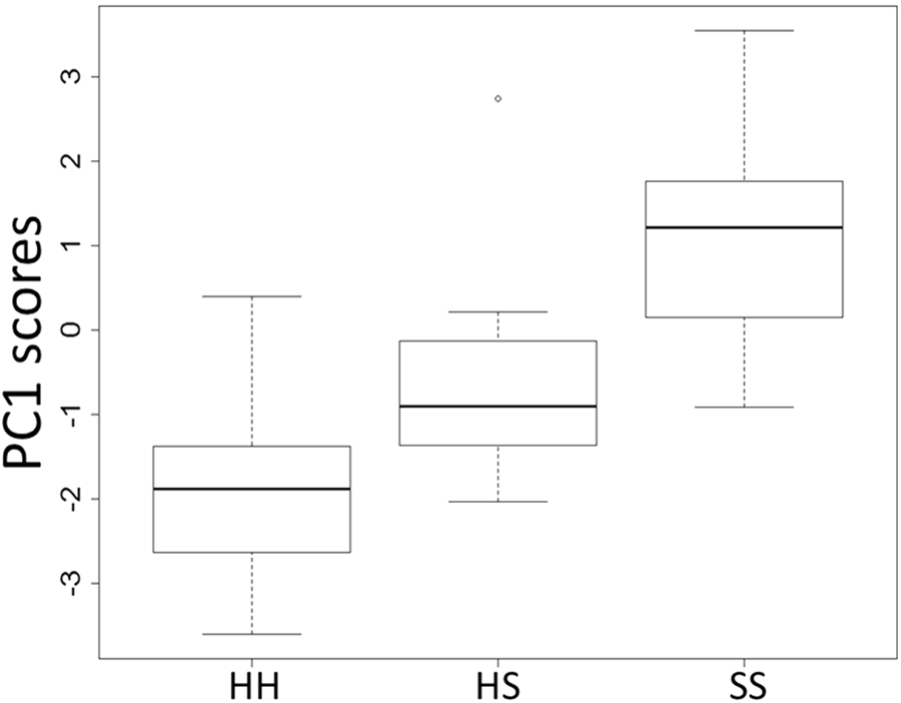
The first axis of the principal component analysis performed on the oxidative status biomarkers was able to discriminate the three study groups (indicated on the x axis by the acronyms HH, HS, and SS). The SS group had significantly higher PC1 scores than both the HS and the HH groups (*P* < 0.01).

Four axes were extracted from the PCA on the individuals without clinical signs sampled at the first sampling period (explaining 26, 20, 17, and 14% of the total variance, respectively). None of them were associated with the occurrence of clinical signs (GLM: *Z* < 1.48, *P* > 0.14). Three axes were extracted from the PCA on the 22 individuals with clinical signs sampled at the first sampling period (explaining 28, 21, and 17% of the total variance, respectively). None of them were associated with the probability of survival (GLM: *Z* < 0.89, *P* > 0.37). Four axes were extracted from a further PCA performed on all the 44 nestlings from the first sampling period (explaining 28, 17, 16, and 12% of the total variance, respectively). None of the axes were associated with the probability of survival (GLM: *Z* < −1.08, *P* > 0.28). However, values of the first PC differed significantly among the three study groups (HH, HS, and SS; Fig. 5). The SS group showed significantly higher PC1 scores than both the HH group (Tukey HSD: *z* = 5.81, *P* < 0.01) and the HS group (Tukey HSD: *z* = 4.45, *P* < 0.01), while the difference between the HH and the HS groups were almost significant (Tukey HSD: *z* = 2.26, *P* = 0.06). This axis indicated that nestlings with high levels of TBARS (loading = 0.425) and GSSG (0.466) had also low levels of GSH (−0.310) and GSH/GSSG ratio (−0.523).

## Discussion

Our study provides the first evidence in a wild vertebrate that the progress of a disease likely caused by a virus is associated with changes in blood oxidative status, and that some biomarkers of oxidative status are associated with both clinical signs and mortality. In addition, males and females did not show any strong differences in the oxidative status.

The analysis of swab samples detected the presence of herpesvirus DNA in most nestlings including individuals without visible clinical signs, indicating that the virus is widespread in this population. However, the quantification of the number of viral DNA did not give a clear indication that clinical signs are related to viral load. Although in this study we found up to 10^6^ to 10^7^ copies of herpesviral DNA in swabs of some infected individuals, amongst which some were found dead from the disease two weeks later, we cannot prove that the clinical signs are uniquely induced by this herpesvirus. For instance, at the first sampling period, one individual showing clinical signs that was classified as “sick” was negative for herpesvirus DNA in both semi-nested PCR and quantitative PCR; in addition, in two other nestlings with clinical signs that were positive by qualitative semi-nested PCR in either cloacal or tracheal swab, viral copies were too low to be detectable by qPCR. Similar outcomes were obtained from the analyses performed on swabs collected from nestlings not showing any visible clinical signs. The lack of a direct association between herpesvirus copies and clinical signs is however not surprising if we consider the dynamics of viral activity and of the appearance of crusts on the body. Crusts take days to appear after the virus replication begins. Thus, it might be that in those sick nestlings that were negative to herpesvirus, the activity of the virus might have already been reduced or repressed by the immune system of the host, but the disappearance of crusts would take longer^57^. Similarly, the virus might have been activated in some healthy nestlings even if the appearance of clinical signs has not occurred yet. Secondly, despite the use of swabs is one of the most commonly used methods in virology, it might be that we could not sample viral particles in all individuals in a standardized manner. This concern is particularly relevant in the case of herpesviruses, because they have specific sites of latency and reactivation. For the human herpes simplex virus 1 (HSV-1), as an example, the site of latency is in the oropharyngeal mucosa^58^, but after activation, the virus becomes evident at mucocutaneous sites^58^. It is therefore possible that we were not able to sample the oropharyngeal mucosa (or the anal mucosa) in all individuals. A third explanation, even if less likely, might be that the visible clinical signs of birds were the result of a combination of diverse pathogens. For instance, a few studies found co-infections between herpesvirus and the bacterium *Chlamydia trachomatis* ^59^, or papillomavirus^60^.

Irrespective of whether the clinical signs were caused by this herpesvirus alone or a co-infection with another pathogen, our results support an association between the visible clinical signs and an unbalanced oxidative status of blood as previously suggested by a meta-analysis on herpesviruses^34^. Nestlings that had higher concentrations of glutathione in the first sampling period had higher probabilities to develop proliferative skin lesions (Fig. 3). Similarly, in a previous study, wild young great tits *Parus major* infected by avian malaria had higher glutathione when compared to uninfected birds^61^. Despite glutathione is a major cell antioxidant^30^, which is decreased during herpesvirus infections in order to maintain an impairment in intracellular redox status essential for the virus replication^62^, the high levels of glutathione associated with the appearance of clinical signs might indicate that nestlings needed to increase synthesis of this non-enzymatic antioxidant in order to compensate for an increased production of reactive oxygen species and oxidative damage during the early stage of viral replication. While previous studies have looked at the relationship between the oxidative status and the survival perspectives, there are to the best of our knowledge no records on the link among pre-existing levels of antioxidants and the probability of developing clinical signs in animal populations. In addition, TBARS values (a measure of oxidative damage to lipids) were associated with survival probabilities of nestlings. Similarly, in nestling great tits *Parus major*, previous work has found a higher glutathione peroxidase activity in those nestlings with lower survival probabilities until fledging^63^, either low or high GPX activity in those nestlings that were less likely to survive^64^, or no relationship between antioxidant capacity or oxidative damage and survival perspective^65^. As can be seen in Fig. 4, high levels of TBARS were associated with higher chances of mortality, but high levels of TBARS were also found in sick but alive nestlings. This is because all the sick individuals showed high levels of TBARS but some of them were still alive when we left the field. However, sick nestlings appeared to be in a critical condition (e.g., body fully covered by crusts, apathy, hyperkeratosis of eyes) and had probably no chance of recovering. As a result, the use of this model is limited to the association between clinical signs and plasma damage to lipids. Although visible clinical signs are sufficient to provide an assessment of the health status, the marker TBARS might still prove useful to assess the effectiveness of a medical treatment of the animals.

We point out that our conclusions are limited to a period of about two weeks and to the fact that oxidative stress biomarkers have been measured only in one tissue, in both erythrocytes and plasma, which might provide information on intra- and extracellular oxidative status^65^. Given that the disease causes extensive damage on the dermal tissue of these birds, it might be valuable to collect biopsies of dermal tissue to test whether the oxidative status in this tissue might be better associated with the occurrence of clinical signs and survival perspectives. However, in our previous meta-analysis, we found that blood-based markers of oxidative status were very sensitive to herpesvirus infection with an effect size comparable to that of other tissues^34^.

The changes that occurred in several metrics of oxidative status over the study period suggest that oxidative stress might be a molecular mechanism underlying the infectious process. For example, superoxide dismutase activity significantly increased in the HS group from the first to the second sampling period. This result suggests that nestlings invested in the up-regulation of superoxide dismutase activity during the early stages of the infection in order to limit spread of damage. Similarly, up-regulation of the superoxide dismutase activity was observed in humans exposed to rotavirus infection^66^. For the non-enzymatic antioxidants, our results showed a strong increase in the glutathione levels in the infected individuals from the first to the second period, when the viral activity has already caused proliferative skin lesions. The up-regulation of the superoxide enzymatic activity during the first phases of the infection may have led to an increase in the circulating glutathione in the latest stage of the infection, although direct evidence in this study is so far limited and warrants further investigations. Conversely to sick nestlings, there was an increase in the oxidized glutathione in the other two groups (Fig. 2). The increase in oxidized glutathione might indicate that cells were experiencing an oxidative challenge, and that there was activation of the glutathione machinery^67^.

In addition, the basal protein oxidative damage expressed as protein carbonyls was very high in sick individuals (SS group) in comparison with the healthy group (HH group), and the carbonylation significantly increased in the HH and the HS group over time. On the contrary, TBARS did not increase from the first to the second period in any of the groups, but only showed a significant and general increase in all the nestlings. In addition, the level of TBARS was very high in individuals with clinical signs (SS group, Fig. 2). The fact that TBARS did not increase in the healthy-looking individuals from the first to the second sampling period, indicates that any increase in damage to lipids might occur only at an advanced stage of the disease and it does not occur simultaneously with the appearance of visible clinical signs. In other words, it might take several days from the appearance of proliferative skin lesions for plasma levels of TBARS to increase.

Outcomes of the models performed on OS biomarkers measured at the first sampling period also supported our conclusions. None of those models could detect any significant differences between the HS and the HH group (which included five more individuals than in the repeated measurement approach), and between the SS and the SD group, therefore indicating that the repeated measurement approach does not suffer from a possible bias caused by the selective disappearance of the dead individuals (or the individuals which have not been found at the second sampling period), possibly because SS and SD nestlings showed similar OS values. Finally, although some biomarkers of oxidative status were associated with clinical signs of the disease or mortality, we could not find a similar pattern in the association of the different biomarkers to the disease. A principal component analysis only showed that HH, HS and SS nestlings differed in the association among TBARS, GSSG, GSH and GSH/GSSG, with SS nestlings having higher TBARS and GSSG and lower GSH and GSH/GSSG in comparison with the HS and the HH groups, respectively. These results were due to the weak correlations among markers as shown by the low variance explained by each single principal component, indicating that a PCA approach is less indicative than a single-biomarker approach in this particular study. The first mortality events of nestling frigatebirds in Grand Connétable natural reserve were recorded in 2005. A monitoring program carried out by the rangers of the natural reserve enabled to estimate a high mortality rate due to the disease, with values that reached about 85 to 95% of nestlings in 2015 (field observations). It is, however, unclear how the virus spreads into the population. Field observations showed that several nestlings did not show any clinical signs even when having many sick neighboring nestlings, suggesting that a vertical transmission of the virus is the more likely scenario of transmission in our population. Previous work on domestic species suggested that vertical transmission is indeed common in herpesviruses^6^. Further research in this direction is clearly needed to better understand the way the virus proliferates in this, as well as, in other wild bird populations.

If the mortality rate remains so high for several years, the disease might have a huge impact on the long-term local recruitment. Different studies have underlined the role of parasites and pathogens as drivers of population dynamics^68, 69^. Only recently, a comparative study pointed out that severe diseases of animal populations might drive to local or global extinction^70^. A recent study showed that there is an active exchange of individuals among the frigatebird populations from Barbuda island, the Abrolhos archipelago and our study area^71, 72^. On the one hand, this might fuel the frigatebird population on Grand Connétable island with new breeding individuals ensuring the viability of the population over the long-term. On the other hand, exchanges of birds between those islands might facilitate the spread of the herpesvirus towards other frigatebird colonies. However, despite the interchange of individuals with other colonies, there are no evidences that the disease has occurred in other frigatebird colonies. The same study also pointed out that this frigatebird population is a key population between the Caribbean and Brazilian colonies of frigatebirds. Furthermore, Grand Connétable island hosts diverse seabird species represented by a huge number of breeding couples (as an example, with about 8,000 couples, the population of Cayenne tern (*Thalasseus eurygnathus*) represents more than 30% of the world population), providing a pool of potential hosts which might enable the persistence of the virus over the long term, and could enable transmission to other species, even if cross-species transmissions are rare events in the case of herpesviruses^6^. It is therefore important to investigate the possibility that these birds are also exposed to other pathogens that might be more transmissible from one species to another, as has been found in some mammalian species in South Africa, where a papilloma virus is spreading through different species^9, 10^.

Environmental factors such as pollution, food availability and food quality are well-known to directly or indirectly affect endocrine mechanisms and decrease immune system in different taxa^73–76^, and specifically in birds^77–80^. It is therefore possible that human activities in French Guiana may have led to a low quality habitat for reproduction and survival that cannot sustain the population. In recent years, the shrimp activity, which generates discards that are an important source of food for frigatebirds, has plummeted in French Guiana, and a previous study indicated that frigatebird couples were not able to feed their chicks after the fishery decline^81^. Food scarcity is a condition to which frigatebirds are particularly vulnerable given that they show the longest period of parental care of any bird species, represented by high energetic needs^82^. Furthermore, since frigatebirds are long lived apex predators which occupy the top of their marine food webs, they are particularly exposed to contaminants^83^. A very recent study has found high levels of mercury (Hg) both in adult and nestling frigatebirds breeding on Grand Connétable island^84^, and since Hg is suspected to aggravate herpes simplex virus 2 infection in mice^85^, the mercury issue is particularly important for this population. One or both of these factors may therefore increase the vulnerability of this particular colony of frigatebirds.

## Conclusions

Our work provides the first evidence in a wild vertebrate that oxidative stress might be one mechanism underlying the impact of a viral disease on individual health and survival. The association between oxidative stress and the occurrence of clinical signs we observed stimulates new studies on the relationship between oxidative stress and viral infections. Our work also points out that there is an urgent need to investigate and deepen our knowledge about the possibility that outbreaks of viral infection in this population could also be the result of local factors such as diet and contaminants, and how this pathogen could spread to other frigatebird colonies as well as to other species, a condition which might endanger the survival of several populations. Finally, this study indicates that tracheal swab samples might be used as a tool to determine whether herpesvirus is active in individuals that do not show any observable clinical signs of the disease.

We finally suggest that further studies should experimentally investigate the relationship between oxidative status and the occurrence of the disease. For example, administration of antioxidants with a consequent decrease in molecular oxidative damage and viral burden would provide evidence in favour of a role of oxidative stress as a molecular mechanism underlying viral activity in wild birds.

## Electronic supplementary material

Supplementary material
