# Supplementary material for: Oxidative stress biomarkers are associated with visible clinical signs of a disease in frigatebird nestlings
Source: Sci Rep. 2017 May 9;7:1599. doi: 10.1038/s41598-017-01417-9 (PMC5431617; doi:10.1038/s41598-017-01417-9)
Supplement: Supplementary file 1 — Supplementary material [file 41598_2017_1417_MOESM1_ESM.doc]

**Supplementary information**

**Oxidative stress biomarkers are associated with visible clinical signs of a disease in frigatebird nestlings**

**Manrico Sebastiano^1*^, Marcel Eens^1^, Hamada Abd Elgawad^2^, Benoît de Thoisy^3^, Vincent Lacoste^3^, Kévin Pineau^4^, Han Asard^2^, Olivier Chastel^5^, David Costantini^1,6,7^**

1. Behavioural Ecology and Ecophysiology group, Department of Biology, University of Antwerp, Universiteitsplein 1, 2610 Wilrijk, Belgium
2. Integrated Molecular Plant Physiology Research, Department of Biology, University of Antwerp, Groenenborgerlaan 171, 2020 Antwerp, Belgium

3. Laboratory of Virus-Host Interactions. Institut Pasteur de la Guyane, French Guiana, France

4. Groupe d'Etude et de Protection des Oiseaux en Guyane (GEPOG), 15 Avenue Pasteur 97300 Cayenne, French Guiana

5. Centre d’Etudes Biologiques de Chizé (CEBC), UMR7372- CNRS/University of La Rochelle, F-79360, France

6. Institute of Biodiversity, Animal Health and Comparative Medicine, School of Life Sciences, University of Glasgow, Graham Kerr Building, Glasgow G12 8QQ, UK

7. UMR 7221, Muséum National d’Histoire Naturelle, 7 rue Cuvier 75231 Paris cedex 05, France

* Corresponding author: [Manrico.Sebastiano@uantwerpen.be](mailto:Manrico.Sebastiano@uantwerpen.be)

**Biomarkers of oxidative stress measurement**

In order to determine the non-enzymatic antioxidant capacity we applied Reversed-Phase HPLC of Shimadzu (Hai Zhonglu, Shanghai) in red blood cells following a previous protocol [^1^](#_ENREF_1). Reduced glutathione (GSH) and oxidised glutathione (GSSG). The ratio between GSH/ GSSG was also calculated and used as a metric of oxidative status [^2^](#_ENREF_2). Briefly, an amount of about 10 μL of red blood cells were put in a 2mL tube to calculate the sample weight. After we froze the sample in liquid nitrogen, we used a magnalyzer at 6000 rpm for 10s to crush the sample into a fine powder and we repeated this step for three times. Then, samples were put in ice and 1mL of 6% metaphosphoric acid (MPA) solution was added. Samples were magnalyzed at 5000rpm for 10s (three times by quickly freezing the samples in liquid nitrogen after every run). Sample were then put in ice and centrifuged for 12min at 14000rpm in a cold centrifuge (4 degrees). After spinning, the supernatant was collected and transferred in a 2mL eppendorf tube in cold. Then, 100 μL of the supernatant were transferred in a white glass vial and we added 300 μL of eluent (2mM KCl, adjusted to pH=2.5 with ortho-phosphoric acid) to measure reduced glutathione. Then another 100 μL of the supernatant were transferred in a dark glass vial and 50 μL of reductant (30mg DTT and 48mg Tris in 1ml aqua distilled water) were added. After storing the samples for 10min in the dark at room temperature, 250 μL of eluens were added to stop the reduction and to calculate total glutathione. Oxidised GSH values are estimated as the difference between the reduced and the total GSH. Concentrations of GSH and GSSG were calculated using a standard curve created by a known concentration of GSH and expressed as μmol/g of fresh weight of red blood cells. The non-enzymatic antioxidant capacity of plasma was quantified using the OXY absorbent test (Diacron International, Grosseto, Italy). This kit quantifies the ability of the antioxidant barrier to cope with the oxidant action of hypochlorous acid (HOCl) using a colorimetric determination. The serum (10 μL) was diluted 1:100 with distilled water. A 200 μL aliquot of a titred HOCl solution was incubated with 5 μL of the diluted serum for 10 min at 37 degrees. Then, 5 μL of the chromogen solution in the kit was added. An alkyl-substituted aromatic amine solubilised in the chromogen is oxidised by the residual HOCl and transformed into a pink derivative. The intensity of the coloured complex, which is inversely related to the antioxidant power, was measured by a spectrophotometer at 490 nm. Calibration was achieved by means of a reference serum able to neutralize 340 mol HOCl/ml. All values were subsequently multiplied x100 to correct for dilution and were expressed as mM HOCl neutralized accordingly to a previous protocol [^3^](#_ENREF_3). Repeatability was estimated with Pearson’s correlation (*r*=0.73).

The activity of antioxidant enzymes, superoxide dismutase (SOD), catalase (CAT) and glutathione peroxidase (GPX) was determined from haemolysates of red blood cells. Red blood cells were homogenized by Magnalyser in 500 µL of phosphate buffer (pH 7.4; 1.15% KCl and 0.02 M EDTA in 0.01 M PBS). After vortexing for 30s, tubes were rapidly put in liquid nitrogen and were then sonicated for 30s in ice. Samples were then magnalysed for 15s at 6500g for three times and then centrifuged at 10000g for 30 min in a cold centrifuge (4 degrees). The supernatant (20 µL) was then transferred in a plate in duplicate. Superoxide dismutase (SOD) activity was determined by measuring the inhibition of nitroblue tetrazolium (NBT) reduction at 560 nm and was expressed as U/mg protein per minute [^4^](#_ENREF_4). Catalase activity (CAT) was assayed in red blood cells by monitoring the rate of decomposition of H_2_O_2_ at 240 nm and was expressed as μmol H_2_O_2_/mg protein per minute [^5^](#_ENREF_5). Glutathione peroxidase (GPX) activity was measured in red blood cells by a spectrophotometric method and was expressed as μmol NADPH/mg protein per minute [^6^](#_ENREF_6). SOD, Spearman’s *ρ*=0.90; CAT, Spearman’s *ρ*=0.95, GPX, Pearson’s *r*=0.84).

Plasma lipid peroxidation was quantified using the Thiobarbituric Acid Reactive Substances (TBARS) assay. Samples were mixed with 0.5 mL of TBA reagent (0.5% (w/v) thiobarbituric acid (TBA) in 20% TCA), vortexed for 30s and incubated at 90°C for 45 minutes. After incubation, samples were cooled in a bath ice to stop the reaction and were centrifuged at 5000rpm for 1 min and the supernatant was transferred in a plate. Absorbance was measured at 532, 600 and 450 nm and amount of MDA equivalents was calculated using the formula 6.45*(A_532_-A_600_) - 0.56*A_450_. Values were expressed as nmol MDA equivalents/g of plasma [^7^](#_ENREF_7). Repeatability calculated with Pearson’s correlation was *r*=0.88.

Finally, to measure protein carbonyl content after samples had been diluted with distilled water in order to have a concentration of 2 mg protein/mL. Then, we added the DNPH reagent (four times the volume of the initial sample) and we incubated the samples in the dark at room temperature for one hour (vortexing the tubes every 15 min). Then, the same volume (as the total volume in the tube: sample + DNPH) of 20% TCA was added to each tube, and tubes were then transferred in ice for 5 minutes. For three times, we centrifuged the samples for 10 min at 10000rpm, we discarded the supernatant, and we re-suspended the pellets in 100 μL of Ethanol/Ethyl Acetate mixture (1:1). After the final wash, protein pellets were re-suspended in 200 μL of guanidine hydrochloride by vortexing and samples were incubated for 15min at 37 degrees with continuous shaking. After final centrifugation at 10000rpm for 10 minutes (at 4 degrees), the supernatant was transferred in a plate. Absorbance was measured at 370nm and the concentration of protein carbonyls was expressed as nmol/mg protein [^8^](#_ENREF_8). Repeatability calculated with Spearman’s correlation was *ρ*=0.97.

**Figure S1: Mean and standard error of the biomarkers of OS at the first sampling period.** Different groups are indicated on the x axis by the acronyms “HH” (n=16), “HS” (n=6), “SS” (n=14), and the “HD” group (n=8). After post-hoc comparisons on the GSSG, GSH/GSSG, CARBONYLS, and TBARS models, plots which share the same letter showed no significant differences.

**
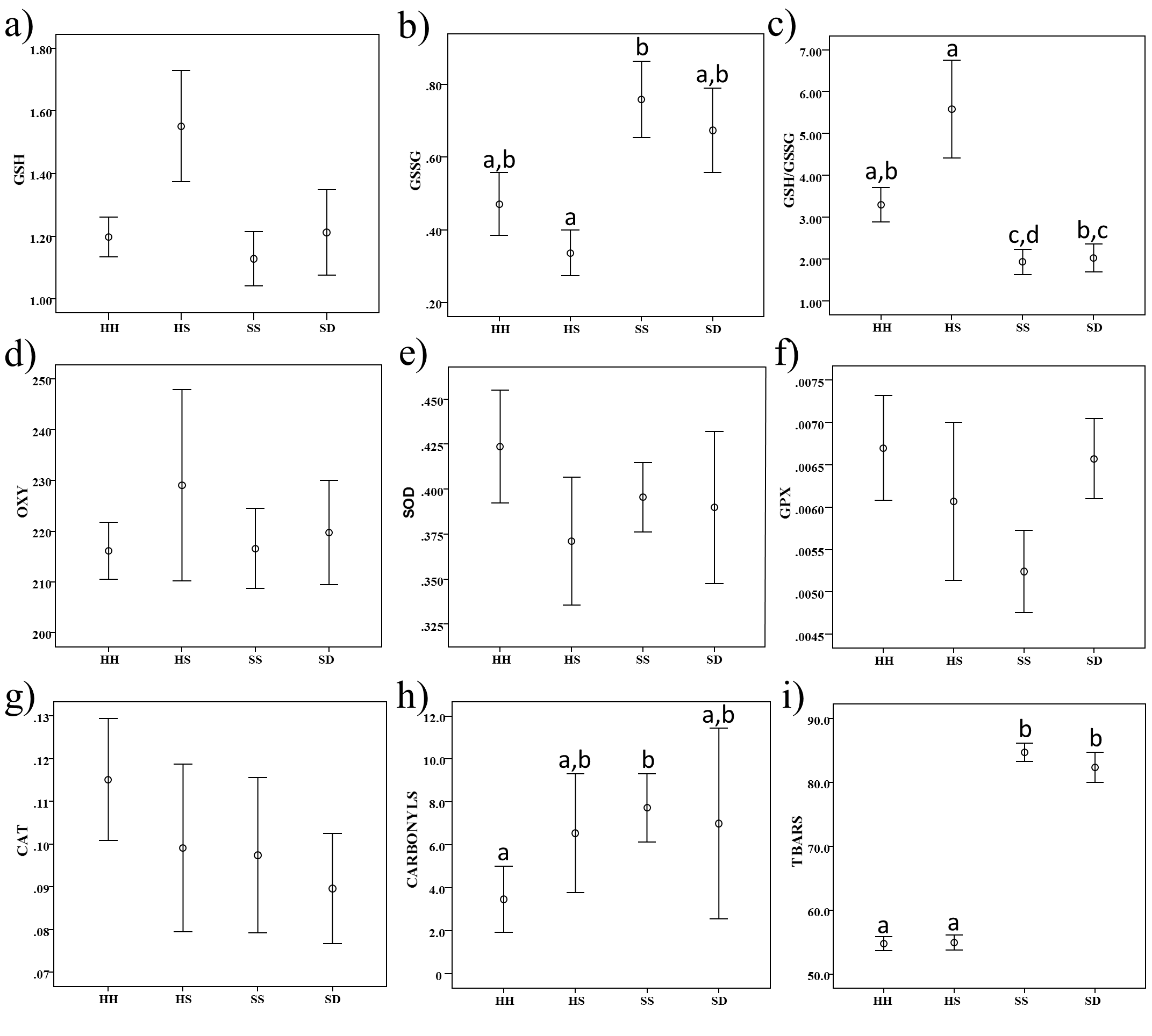
**

**Table S1: Results of the semi-nested and quantitative PCRs on tracheal and cloacal swabs of frigatebird nestlings.**

|  |  | **First sampling period** | | | |
| --- | --- | --- | --- | --- | --- |
|  |  | **Tracheal swabs** | | **Cloacal swabs** |  |
| **Individual** | **Clinical signs** | **Nested PCR** | **qPCR** | **Nested PCR** | **qPCR** |
| 1 | absent | negative | undetected | positive | undetected |
| 2 | absent | negative | undetected | negative | undetected |
| 3 | absent | negative | undetected | negative | undetected |
| 4 | absent | negative | undetected | positive | <40 |
| 5 | absent | negative | undetected | negative | undetected |
| 6 | absent | positive | <40 | positive | undetected |
| 7 | absent | positive | 1.8*10^4^ | positive | undetected |
| 8 | absent | positive | 2.7*10^4^ | positive | <40 |
| 9 | absent | positive | 2.8*10^5^ | positive | undetected |
| 10 | absent | positive | 1.1*10^4^ | positive | 106 |
| 11 | present | positive | undetected | positive | undetected |
| 12 | present | negative | undetected | negative | undetected |
| 13 | present | positive | undetected | negative | undetected |
| 14 | present | negative | undetected | positive | 168 |
| 15 | present | positive | 1.1*10^7^ | negative | undetected |
| 16 | present | positive | 3.2*10^6^ | positive | <40 |
| 17 | present | positive | 1.1*10^7^ | positive | 267 |
| 18 | present | positive | 2.8*10^5^ | positive | undetected |
| 19 | present | positive | <40 | positive | <40 |
| 20 | present | positive | 2.7*10^6^ | positive | 162 |
|  |  | **Second sampling period** | | | |
|  |  | **Tracheal swabs** | | **Cloacal swabs** |  |
| **Individual** | **Clinical signs** | **Nested PCR** | **qPCR** | **Nested PCR** | **qPCR** |
| 21 | absent | positive |  | positive |  |
| 22 | absent | positive |  | negative |  |
| 23 | absent | positive |  | negative |  |
| 24 | absent | positive |  | negative |  |
| 25 | absent | positive |  | positive |  |
| 26 | absent | positive |  | positive |  |
| 27 | absent | positive |  | negative |  |
| 28 | absent | positive |  | positive |  |
| 29 | absent | positive |  | negative |  |
| 30 | absent | positive |  | negative |  |
| 31 | present | positive |  | negative |  |
| 32 | present | positive |  | negative |  |
| 33 | present | negative |  | negative |  |
| 34 | present | negative |  | negative |  |
| 35 | present | positive |  | negative |  |
| 36 | present | negative |  | negative |  |
| 37 | present | positive |  | negative |  |
| 38 | present | negative |  | negative |  |
| 39 | present | negative |  | positive |  |
| 40 | present | negative |  | negative |  |
| 41 | present | positive |  | negative |  |

**References :**
